# Effects of Radial Extracorporeal Shockwave Therapy on Piriformis Syndrome: A Single-Case Experimental Design

**DOI:** 10.7759/cureus.61873

**Published:** 2024-06-07

**Authors:** Syoya Nakanishi, Masahiro Tsutsumi, Kengo Kawanishi, Makoto Wada, Shintatou Kudo

**Affiliations:** 1 Graduate School of Health Sciences, Morinomiya University of Medical Sciences, Osaka, JPN; 2 Inclusive Medical Sciences Research Institute, Morinomiya University of Medical Sciences, Osaka, JPN; 3 Orthopaedics, Wada Orthopaedic Clinic, Osaka, JPN

**Keywords:** radial extracorporeal shockwave therapy, sciatic nerve, piriformis hardness, peripheral entrapment neuropathy, piriformis syndrome

## Abstract

The effects of radial extracorporeal shockwave therapy (rESWT) on piriformis syndrome were investigated using a single-case study design.

This study used an AB single case with a follow-up phase design. The baseline phase consisted of general physical therapy, including piriformis stretching, while the experimental phase consisted of rESWT in addition to general physical therapy. A man in his 70s diagnosed with piriformis syndrome participated in the study. The Numerical Rating Scale (NRS) score, piriformis hardness, and cross-sectional area of the sciatic nerve were measured to determine the effectiveness of the intervention. The baseline and experimental phases were compared using a binomial distribution based on the celeration line.

The NRS score, piriformis hardness, and cross-sectional area of the sciatic nerve were significantly decreased in the experimental phase compared to the baseline phase (NRS, p<0.001; piriformis hardness, p<0.001; cross-sectional area of the sciatic nerve, p<0.001). This effect was carried over to the follow-up phase for all variables.

rESWT for piriformis syndrome improved the clinical symptoms, piriformis hardness, and cross-sectional area of the sciatic nerve. However, these results are exploratory and require further validation in future clinical trials.

## Introduction

Piriformis syndrome is an entrapment neuropathy caused by compression exerted by the piriformis on the sciatic nerve [1]. This compression causes pain in the lower back and buttocks, radiating along the posterior femur and aligning with the distribution of the sciatic nerve [2]. Physical therapy often stretches the piriformis to manage piriformis syndrome [1,2]. However, pharmacotherapy and physical therapy showed a symptom resolution rate of 51.2% in 250 patients diagnosed with piriformis syndrome [3]. Therefore, better treatment options are needed.

Radial extracorporeal shockwave therapy (rESWT) is a new, noninvasive treatment for entrapment neuropathy. Rigorous randomized controlled trials have demonstrated the utility of rESWT in addressing chronic back pain [4], knee osteoarthritis [5], plantar fasciitis [6], lateral epicondylitis [7], and carpal canal syndrome [8-10]. Moreover, some reports have indicated the potential efficacy of rESWT in carpal tunnel syndrome, an entrapment neuropathy [8-10]. Regarding the impact of rESWT on piriformis syndrome, one previous study [11] has evaluated its efficacy using the Visual Analogue Scale (VAS) and SF-36; however, it remains unclear how rESWT affects the pathophysiology of piriformis syndrome.

This study aimed to investigate the effect of rESWT on piriformis syndrome by adding piriformis hardness and sciatic nerve cross-sectional area, which may be affected by rESWT, as outcomes. We used a single-case experimental design that is appropriate for preliminary intervention studies. We hypothesized that rESWT would improve piriformis hardness, cross-sectional area of the sciatic nerve, and associated clinical symptoms.

## Case presentation

Design

This study used an ABA single-case experimental design, with A as the baseline phase and B as the experimental phase. Each phase consisted of eight sessions (one month) of physical therapy of 20 minutes each. The interventions were conducted twice a week for three months. Based on previous studies, the baseline phase consisted of general physical therapy including stretching of the piriformis and strength training of the lumbar and hip joints [1,2]. In the experimental phase, rESWT was performed in addition to general physical therapy.

Procedural changes

Physical therapy was terminated at the end of the experimental phase because the participant's symptoms of pain and heaviness from the left buttock to the posterior thigh improved. Therefore, general physical therapy could not be administered in the second baseline phase. The ABA single-case design was then changed to an AB single-case design with a follow-up phase design [12]. The follow-up phase was conducted once every two weeks, and the variables described below were measured.

Participant

He was a man in his 70s, diagnosed with piriformis syndrome by an orthopedic surgeon. The diagnosis was based on criteria such as buttock pain as the main symptom, radiating pain in the lower extremities, tenderness in the piriformis, and a positive piriformis tension test [13]. Participant's data were collected at an orthopedic clinic in Hirakata, Osaka, Japan. The participant began to have symptoms one week before visiting our clinic, was checked at our clinic, and started physical therapy on the same day. The participant was diagnosed with knee osteoarthritis six months prior and was receiving weekly intra-articular knee injections (purified sodium hyaluronate) and nonsteroidal inflammatory drugs. The symptoms included pain and heaviness from the left buttock to the posterior thigh, and the pain was elicited by rising, sitting, walking, coughing, and sneezing. In an orthopedic examination [1], the Friberg and active piriformis tests were positive, and the Pace, Beatty, and neurodynamic stretching tests were negative. Neurological examinations revealed normal sensory and tendon reflexes. Manual Muscle Testing included hip flexion (4/4) (left/right), hip extension (4/4), hip abduction (4/4), hip adduction (4/4), knee extension (4/5), and knee flexion (4/4). Tenderness was observed in the left piriformis, conjoint tendon of the obturator internus, gemellii superior and inferior, quadratus femoris, and sciatic nerves. Following the Declaration of Helsinki, the purpose, methods, management, and use of the study results were fully explained to the participant, and his informed consent was obtained in writing.

Intervention

rESWT (MASTERPULS MP100; STORZ MEDICAL, Tokyo, Japan) was performed in the experimental phase. A D20-T transmitter (MASTERPULS MP100, STORZ MEDICAL) was used. The irradiated position was the side-lying position with the piriformis extended, hip flexion at 120°, adduction at 30°, and external rotation at 50°, as reported by Gulledge et al. [14]. The irradiation site was positioned where the sciatic nerve entered the deep part of the piriformis and was confirmed using ultrasound imaging. After the coupling gel was applied, irradiation was performed as follows: 4.0 bar, frequency of 15 Hz, and 2000 shocks. In this case, the piriformis was 3-5 cm deep in the skin. The irradiation depth for D20-T was 5 cm. Therefore, the rESWT was pressed down so that it sank approximately 2 cm to allow irradiation of the deep sciatic nerve.

Outcomes

**Figure 1:**
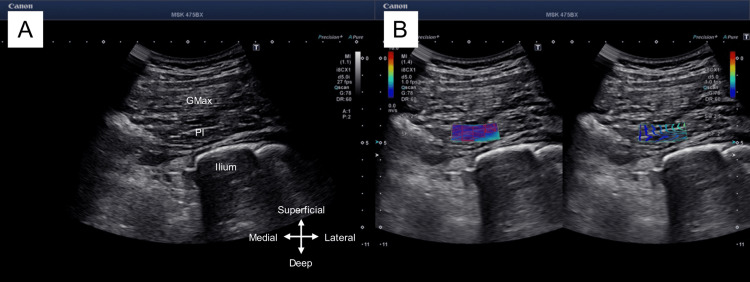
Measurement of piriformis hardness. The piriformis is described (A), the region of interest for shear wave elastography was set within the piriformis (B), and the shear wave velocity (m/s) in the piriformis was measured. GMax: gluteus maximus; PI: piriformis

**Figure 2:**
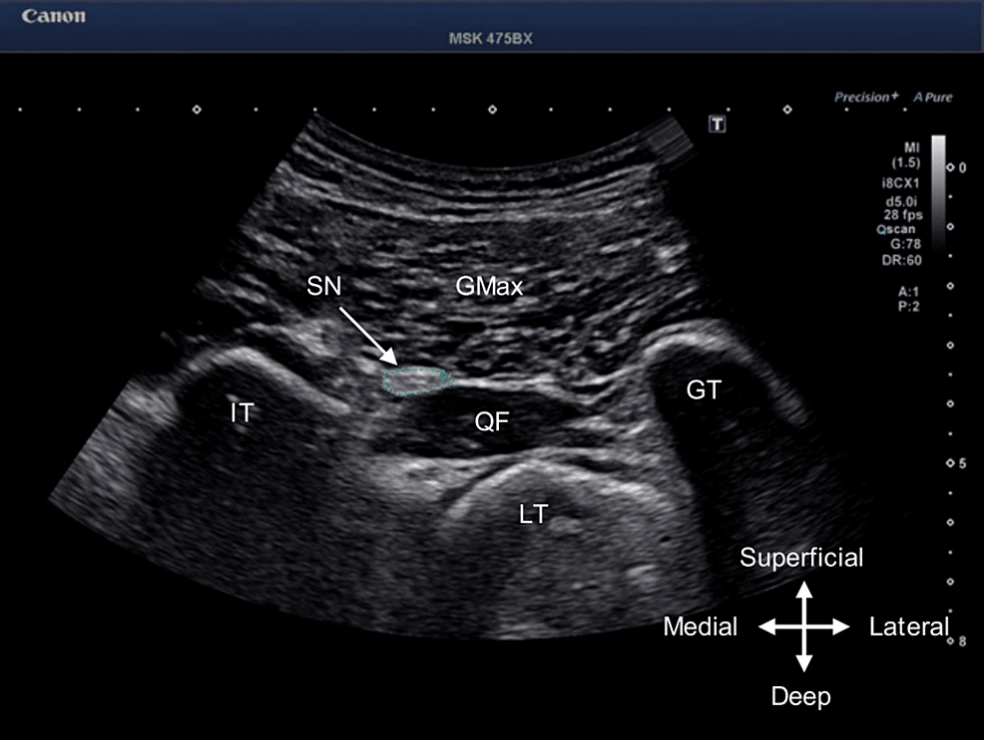
Measurement of the cross-sectional area of the sciatic nerve. The cross-sectional area of the sciatic nerve between the quadratus femoris and the gluteus maximus was measured at the level of simultaneous visualization of the ischial tuberosity and greater trochanter. GMax: gluteus maximus; QF: quadratus femoris; IT: ischial tuberosity; GT: greater trochanter; LT: lesser trochanter; SN: sciatic nerve (arrows)

Assessments were conducted at the end of each physical therapy session to determine the effects of the intervention. The degree of pain during the exercise was measured using a Numerical Rating Scale (NRS). The NRS is a graded scale that indicates the current level of pain on a scale of 0-10, with 0 indicating no pain and 10 indicating the greatest imaginable pain [15]. The effect on piriformis syndrome was determined by measuring the hardness of the piriformis and the cross-sectional area of the sciatic nerve. Measurements were performed in the supine position (hip flexion, 45°; knee flexion, 90°), as described in previous studies [16]. An 8 MHz transducer (PVT-375BT; Canon Medical Systems Inc., Tochigi, Japan) with an ultrasound imaging system (Aplio i700; Canon Medical Systems Inc.) was used. The region of interest in shear wave elastography was set within the piriformis, and shear wave velocity was used to measure piriformis hardness (Figure 1). The cross-sectional area of the sciatic nerve between the quadratus femoris and the gluteus maximus was measured at the level of simultaneous visualization of the ischial tuberosity and greater trochanter (Figure 2). For the unaffected side, piriformis hardness and cross-sectional area of the sciatic nerve were measured only for the first time in the baseline phase. The measurement and intervention were performed by the same person who had experience in performing it more than once prior to the study.

Statistical methods

The central partitioning method was used to obtain the celeration line (CL) from the baseline phase, and a binomial distribution was used to examine the upper or lower number of each variable compared with the CL extended to the experimental phase. The baseline and experimental phases calculated the Tau-U to confirm the effect size. Tau-U is the effect size based on the overlap of data considering the trend in the baseline phase [17]. The evaluation criteria for Tau-U by Vannest and Ninci [17] are as follows: 0.80-1.00, very large change; 0.60-0.80, large change; 0.20-0.60, moderate change; and 0.00-0.20, small change. Statistical analyses were conducted using R.4.0.2 (company name, city, country) for the binomial distribution and the single-case effect size calculator version 0.7.2.9999 (company name, city, country) for Tau-U. The threshold for significance was set at p<0.05.

Results

The participant completed the entire program for the duration of both the baseline (general physical therapy) and experimental (rESWT) phases. The participant was unable to perform the second baseline phase (general physical therapy) because his symptoms improved as he completed the experimental phase. Therefore, the design was changed to an AB single-case design with a follow-up phase, and comparisons between the experimental and follow-up phases were performed using visual analysis. The baseline and experimental phases of each variable were measured during the entire period, whereas the follow-up phase was measured once every two weeks. The most commonly reported side effects were transient pain and skin redness after irradiation in rESWT. These side effects disappeared naturally over time. The Friberg, Bonnet, and active piriformis tests were negative at the end of the experimental phase, and tenderness resolved in the left piriformis, internal obturator muscle, quadriceps femoris muscle, and sciatic nerve.

**Figure 3:**
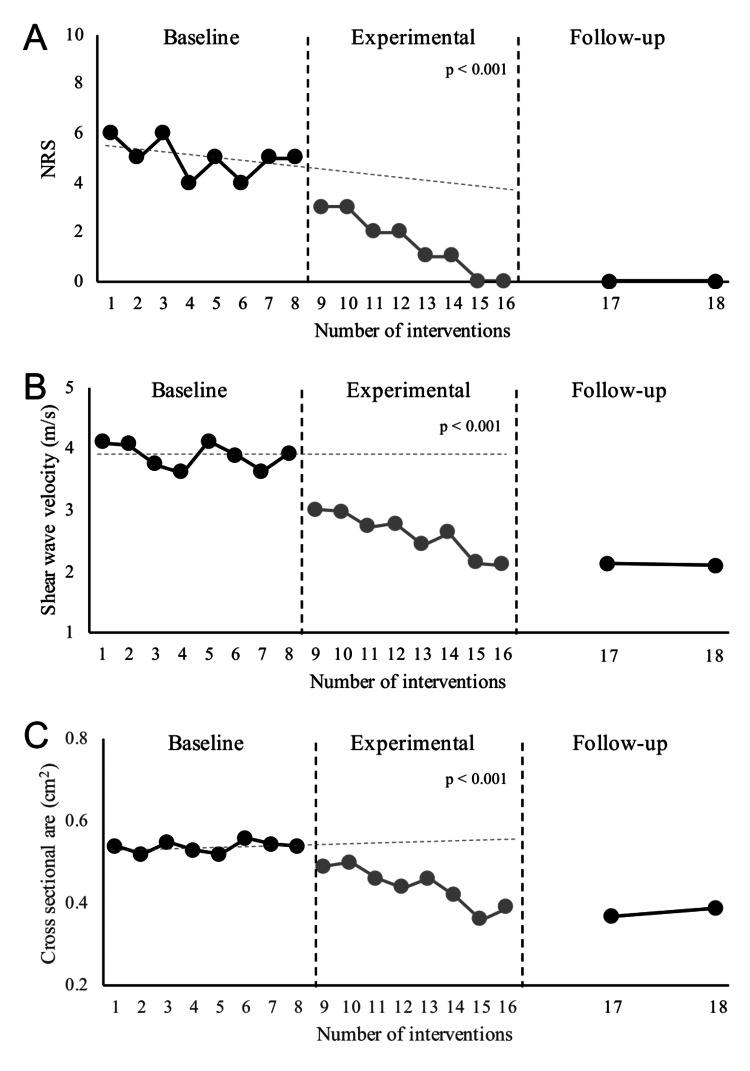
Analysis results for each variable. Dotted lines indicate the celeration line. The NRS (A), piriformis hardness (B), and cross-sectional area of the sciatic nerve (C) significantly decreased in the experimental phase with rESWT compared to the baseline phase (p<0.001). This effect persisted to the follow-up phase. NRS: Numerical Rating Scale; rESWT: radial extracorporeal shockwave therapy

Piriformis hardness and cross-sectional area of the sciatic nerve were higher on the affected side than on the unaffected side at the start of the study (piriformis hardness: unaffected side vs. affected side, 2.02 m/sec vs. 4.02 m/sec; cross-sectional area of the sciatic nerve: 0.38 cm^2^ vs. 0.54 cm^2^). The NRS, piriformis hardness, and cross-sectional area of the sciatic nerve were significantly decreased in the experimental phase (rESWT) compared to those in the baseline phase (NRS: mean value of the baseline phase=5.00, mean value of the experimental phase=1.50, p<0.001, Tau=1.00; piriformis hardness: 3.89 m/sec, 2.61 m/sec, p<0.001, Tau=0.91; cross-sectional area of the sciatic nerve: 0.54 cm^2^, 0.44 cm^2^, p<0.001, Tau=1.00) (Figure 3). The effect was carried over to the follow-up phase for all variables (NRS: mean value of the experimental phase=1.50; mean value of the follow-up phase=0.00; piriformis hardness: 2.61 m/sec, 14 m/sec, cross-sectional area of the sciatic nerve: 0.44 cm^2^, 0.38 cm^2^).

## Discussion

We performed rESWT in a patient with piriformis syndrome. The results showed that the NRS scores, piriformis hardness, and cross-sectional area of the sciatic nerve improved in the experimental phase compared with the baseline phase, and the effect was sustained during the follow-up phase.

rESWT for entrapment neuropathy has been shown to reduce VAS, Boston Carpal Tunnel Questionnaire (BQ), and sensory nerve action potential distal latency [8-10]. In patients with piriformis syndrome, a randomized controlled trial of rESWT versus corticosteroid injection showed that both groups had improved VAS [11]. The present study demonstrated not only a reduction in pain but also a reduction in piriformis hardness and cross-sectional area of the sciatic nerve with respect to the effect of rESWT on piriformis syndrome.

The pathophysiology of entrapment neuropathy involves ischemic damage caused by nerve compression and subsequent microvascular dysfunction, resulting in intraneural edema [18]. Thus, the increased cross-sectional area of the sciatic nerve before the intervention may be interpreted as neural edema, and decreased piriformis hardness by rESWT may improve sciatic nerve edema, resulting in a decreased cross-sectional area.

The results of this study are exploratory and should not be generalized beyond the sample. However, the findings suggest that physical therapy in piriformis syndrome has been focused on stretching the piriformis; however, rESWT may be another noninvasive and effective intervention method. Intervention studies should be conducted based on the results of this study.

This study has several limitations. First, the design was changed to an AB single-case experiment with a follow-up phase design; therefore, we could not statistically examine the experimental and follow-up phases. Second, the follow-up phase was not controlled for internal validity history and maturation because the numbers did not return to baseline. Third, because this study used an ABA single-case experimental design, the generalizability should be interpreted with caution. For example, the rESWT transmitter used in this study can irradiate a depth of 5 cm. It is unclear whether the same effect can be achieved in patients with thicker subcutaneous tissue in the pudendal region, because irradiation may not reach the sciatic nerve. Fourth, the mechanism of rESWT presented in the discussion section is speculative. Kisch et al. [19] reported that rESWT increases soft tissue blood flow, suggesting that rESWT may also directly improve the function of the sciatic nerve microvasculature and reduce its cross-sectional area. We should measure the blood flow around the sciatic nerve in a future study to elucidate the effect of rESWT on piriformis syndrome. Fifth, the follow-up duration was shorter, and fewer measurements were taken. Therefore, it is unclear whether the effect of rESWT will persist over a further period.

## Conclusions

We tested the efficacy of rESWT using a single-case experimental design in a man in his 70s diagnosed with piriformis syndrome. rESWT improved the clinical symptoms of piriformis syndrome, piriformis hardness, and the cross-sectional area of the sciatic nerve, and the effect was significant and sustained. The results of this study are exploratory, and the efficacy of rESWT for piriformis syndrome should be tested in future clinical trials. Also, there are also treatments for piriformis syndrome, such as corticosteroid injections, and the combined effect of these treatments and rESWT should be tested in the future.
